# Total Methane and CO_2_ Emissions from Liquefied Natural Gas Carrier Ships: The First Primary Measurements

**DOI:** 10.1021/acs.est.2c01383

**Published:** 2022-06-14

**Authors:** Paul Balcombe, Dalia A. Heggo, Matthew Harrison

**Affiliations:** † School of Engineering and Material Sciences, Queen Mary University of London, London E1 4NS, U.K.; ‡ SLR International Corporation, 22118 20th Ave SE, Bothell, Washington 98021, United States

**Keywords:** LNG carrier ships, methane emissions, engine slip, greenhouse gas emissions, natural gas supply chain, bottom-up measurement, FTIR and OGI measurement

## Abstract

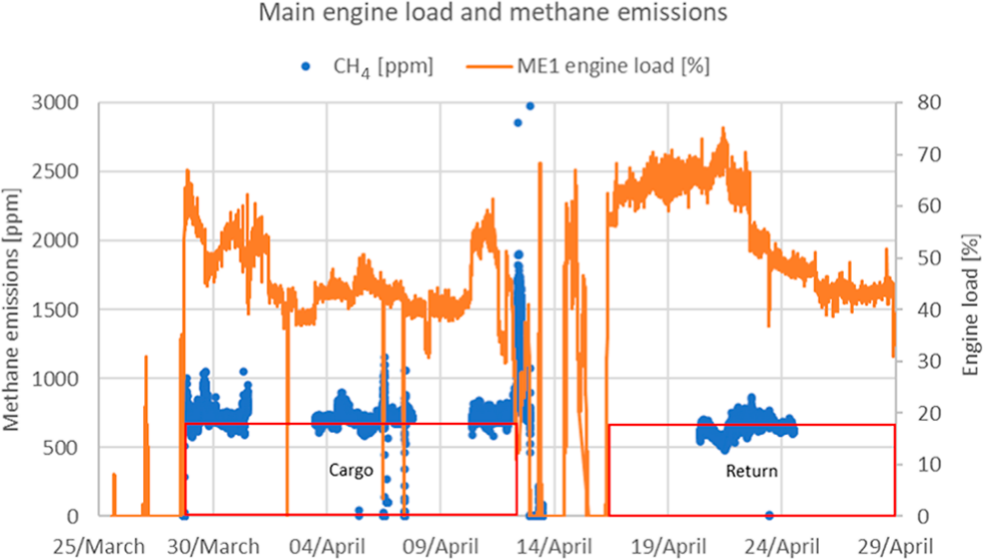

Mitigating methane emissions is vital in meeting global climate targets, but there is a lack of understanding of emissions and abatement opportunities to enable this. The natural gas supply chain is a key emission source, where methane emissions from liquefied natural gas (LNG) shipping have until now not been directly measured. This study provides the first measurement and modeling of total methane and CO_2_ emissions from an LNG carrier on a round trip voyage from the USA to Belgium and back, including loading, laden voyage, unloading, and ballast voyage, measuring emissions from exhaust stacks, vents, and fugitives. Venting and fugitive emissions were extremely low, contributing less than 0.1% of total greenhouse gas emissions. CO_2_ emissions from fuel usage were also lower than previous estimates due to improved efficiencies in modern engines and ship design. However, methane slip through the engines were higher than those in prior studies, averaging 3.8% across all engines: equating to 0.1% of delivered LNG. Generator engines are not typically included in emissions analyses but were the key cause of methane emissions. Engines exhibited higher methane slip rates at low loads, and optimized operation could reduce slip rates by half. More measurement studies are now needed to better understand fleet emissions and enable cost-effective mitigation strategies.

## Abstract 2

This study is the first measurement and modeling of total methane and CO_2_ emissions from an LNG carrier ship to better understand natural gas supply chain emissions. CO_2_ emissions were substantially lower than previous estimates, but methane slip was higher, particularly from generator engines.

## 1 Introduction

Methane is the second most prevalent greenhouse gas (GHG), contributing a quarter of today’s manmade warming,^1^ and methane mitigation is vital in meeting a 1.5 or 2 °C global temperature limit.^2^ Methane emissions arise from several sources including oil and gas, agriculture, and wetlands, and over the last 10 years, we have seen a rapid development in our understanding across the natural gas supply chain, including many primary measurement studies in the USA.^3^ Both methane and CO_2_ emissions have been found to be highly variable,^4^ particularly across supply chain stages,^5^ regions,^6^ and operators.^7^ However, there remains substantial gaps in our understanding of methane emissions from many regions and particularly relating to liquefied natural gas (LNG) transport.

The international LNG trade is a rapidly growing part of the natural gas industry and may increase further as the move to decarbonize national energy systems requires the decommissioning of more carbon intensive domestic supply (e.g., coal for electricity). LNG may offer reduced GHG emissions when compared to other sources such as coal for electricity or diesel as a marine fuel, but the benefit is dependent on limiting methane emissions that offset the CO_2_ advantage for natural gas.

To date, there have been no direct measurements of total methane and CO_2_ emissions from LNG shipping. While some studies have conducted measurements of methane emissions from marine engine slip,^8−11^ none have published measurements of total methane emissions, which include fugitives (unintentional leaks, typically from seals or equipment connections) and venting emissions (intentional emissions via dedicated outlets to atmosphere) from the onboard LNG and vapor handling plant. There is an urgent need to understand the GHG emission profiles of imported LNG to meet national and international climate targets and corporate climate strategies.

This paper begins to fill this critical knowledge gap by conducting the first total methane and CO_2_ emissions measurement campaign for an LNG carrier. The aim of this study is to quantify total methane and CO_2_ emissions associated with an LNG carrier using direct measurement to identify the key contributors to total emissions. A measurement campaign was carried out over a full roundtrip voyage from loading, the cargo-laden voyage, unloading, and the return ballast voyage. The measurements are used to develop a multiparametric emissions model of LNG shipping to estimate total GHG emissions. The study also seeks to shed light on the best methods to directly measure methane emissions from LNG carriers that can be employed in future studies or the retrofit of onboard continuous emissions monitors.

## 2 Methodology

### 2.1 LNG Carrier

The GasLog Galveston LNG carrier (LNGC) was chosen for measurement due to the WinGD XDF low-pressure dual-fuel (LPDF) two-stroke engine used for propulsion. The ship was built in 2021, and the measurement campaign was the ship’s second voyage. LPDF two-stroke gas engines are currently the most popular for new ship builds, and to date, there has been no published direct measurements of uncombusted methane in engine exhausts (methane slip) while in operation,^12^ which is consequently the key to estimating fleet emissions in the future. The ship houses two dual-fuel main engines for propulsion and four dual-fuel generator engines to produce power for other ship demands:

- Main engine 1 (M1): WinGD W5X72DF two-stroke, 11,530 kW
- Main engine 2 (M2): WinGD W5X72DF two-stroke, 11,530 kW
- Generator engine 1 (G1): Hyundai HiMSEN 8H35DF four-stroke, 3840 kW
- Generator engine 2 (G2): Hyundai HiMSEN 6H35DF four-stroke, 2880 kW
- Generator engine 3 (G3): Hyundai HiMSEN 6H35DF four-stroke, 2880 kW
- Generator engine 4 (G4): Hyundai HiMSEN 8H35DF four-stroke, 3840 kW

The main engines were typically operated at 60 rpm, and the generators were typically operated at 720 rpm. Further information on the engine operational conditions can be found in the Supporting Information. The main engines are designed for dual-fuel operation: the engines run on either gas or in the diesel mode. In the “gas mode”, boil-off gas (BOG) from LNG cargo tanks is heated and injected into the engine at low pressure, along with a small amount of pilot diesel fuel (0.5–1.5% of the total energy consumption) to facilitate ignition.

LNG cargo is loaded from the terminal into the vessel’s storage tanks while at berth using loading arms. The Galveston’s four insulated cargo tanks collectively hold a maximum of 171,000 m^3^ LNG, stored at near atmospheric pressure and approximately −160 °C. The containment system is a membrane type “Mark III Flex+”, which has a rated maximum boil-off generation of 0.07% of the cargo per day.^13^

During the voyage, heat ingresses into the storage and LNG gradually vaporizes within the cargo tanks, creating BOG. To avoid venting of the tanks or pressure buildup, BOG is removed from the tanks and used as fuel for the propulsion and generator engines. If there is insufficient demand for BOG from the engines, BOG can either be sent to a reliquefaction facility and sent back to the storage tanks or combusted (flared) in the gas combustion unit (GCU).

### 2.2 Voyage

A summary of the voyage is presented in the Supporting Information, Table S1. The researchers boarded the ship on March 25, 2021, while the ship was anchored off the port of Corpus Christi, USA. The ship docked and loaded the cargo in Corpus Christi on 27th March and began the laden voyage across the Atlantic on 28th March. The laden voyage lasted 16 days until the ship docked in Zeebrugge, Belgium on 13th April to discharge the cargo. Unloading began on 13th April, and the returning ballast voyage to USA began on the 14th. The ship stopped to refuel with diesel in Portland, UK, on 15th April, and the ballast voyage lasted another 13 days to reach Corpus Christi on 28th April. For further details on the voyage, please see Supporting Information Table S2.

### 2.3 Measurement Setup

An inventory of all potential sources of methane and CO_2_ emissions from the ship across different operational modes was developed in conjunction with the ship operators and is summarized in the Supporting Information (Table S3). Emissions are categorized as exhausts, vented emissions, and fugitive emissions, of which the measurement setup is described in this section.

#### 2.3.1 Exhaust Monitoring: Continuous Emission Monitoring System

Two extractive Fourier transform infrared (FTIR) continuous emission monitoring systems (CEMSs) were temporarily installed to measure emissions concentrations from seven sources: two main engines, four generator engines, and the GCU. Exhaust gas emission concentrations of methane, CO_2_, O_2_, and water were continuously measured using the two CEMSs: at any one time, two out of the seven exhausts (2× main engine, 4× generators, and 1× GCU) were being monitored. Typically, one main engine and one generator were being monitored at a given time. The sample probe/line was periodically switched from one exhaust to another during the study period to ensure the capture of a representative emission profile across all stacks.

To ensure consistent FTIR performance throughput the testing period, the FTIR system was calibrated with reference gases once per day. Methane and CO_2_ at known concentrations were used to assess the FTIR output readings. The calibration results must be within ±5% of the actual value to validate results as per US EPA Method 320.^14^

#### 2.3.2 Vents

Vented emissions arise from the dedicated vent masts, as well as from maintenance activities where equipment is purged and depressurized prior to breaking connections. There are four vent masts connected to the LNG cargo tanks and one additional vent connected to the engine room. All vent masts are equipped with gas concentration monitors, and the forward mast is equipped with an inline flowmeter. From consultation with the ship operators, venting from the masts was expected to occur rarely, if at all, during the ship operation. If a venting event occurred, a procedure was in place to ensure that the measurement team would receive advance notice from the ship crew and monitor the vent in two ways:

- The forward mast vent flowmeter records the vented volume flowrate
- The event would be recorded by the measurement team using a portable optical gas imaging (OGI) camera.

Only one of the five vent masts (the forward mast, which is common to the vapor main and all cargo tanks) had a flowmeter installed. Therefore, it was agreed that should any vented emissions occur, we would receive prior notification from the operators, as well as use the ship’s gas detection system and finally periodic spot checks of the masts using the OGI camera fixed on a tripod to check for unintended methane leakage. Two OGI cameras were used simultaneously: the FLIR GF320x and the Opgal EyeCGas 2.0.

Venting emissions associated with maintenance activities were monitored, and an inventory was created of all scheduled maintenance activities. Such activities included testing of the vent control valve and checking filters. Each of these activities was monitored using an OGI camera, and the emissions were estimated via a volumetric calculation based on equipment/pipework volumes and operating pressures.

Ship-side methane emissions associated with loading and unloading included any emissions associated with connecting the loading arms, as well as methane slip and venting emissions. Loading arm connections were monitored during connection and disconnection using an OGI camera, and emission volumes were recorded where identified.

Note that engine crankcases vent directly into the funnel of the ship, and we relied on the gas concentration alarm (at 4% vol/vol) that was installed in the vent line to determine the presence of methane. The concentration meters did not alarm at any point for any of the lines, and consequently, emissions were assumed to be negligible.

#### 2.3.3 Fugitive Emissions

Fugitive emissions may occur from any piece of gas-handling equipment, especially via connections, seals, or threads. The facilities onboard the ship with gas-contacting equipment are as follows:

- LNG cargo containment system and associated pipework above the deck (including vents);
- the compressor room;
- the engine room (main and generator engines, GCU, and fuel delivery system);
- the reliquefaction facility; and
- the loading/unloading manifolds.

To detect the presence of fugitive emissions, walking OGI surveys were conducted across all facilities. Walking surveys were mostly conducted daily, where one of the above areas was the focus each day. Each area was surveyed around five times over the voyage.

The main and trunk deck cargo areas, the compressor room, and engine rooms were visited most frequently as these were continuously in operation. The reliquefaction facility did not operate at all during our voyage due to the absence of surplus BOG and so was only visited twice to screen for fugitive emissions as the equipment was not handling gas while idle. The loading and unloading manifolds were screened during use (before, during, and immediately after the loading and unloading operations).

During the walking survey, OGI cameras were used for detection. Each piece of gas-handling equipment was surveyed and recorded as leak/no leak. If a leak was qualitatively observed using OGI, the ship operators were notified. The leak emission rate was quantified before repair using the Bacharach Hi-Flow sampler. Note that the majority of equipment being surveyed was indoors, besides the above deck cargo containment system. The outdoor surveys were only conducted in clear weather conditions, and wind speeds were not considered to negatively impact the survey given that all equipment being surveyed was within a 3 m distance.

#### 2.3.4 Ancillary Data

Along with emissions measurement data collection, ancillary data from the ship’s operating system were collected for the duration of the voyage, to develop a multiparametric model of emissions from the voyage. Data were collected at 1 min intervals to match the emissions measurement for the following:

- Engine load (×6 engines)
- Engine BOG consumption (×6 engines)
- Engine diesel consumption (for each main engine and total diesel consumption for generators)
- Engine exhaust temperature (×6 engines)
- Engine gas/diesel mode (×6 engines)
- Ship speed
- Ambient temperature and pressure conditions
- Cargo volume (×4 tanks)
- Cargo tank pressures and temperatures (×4 tanks)
- Vent mast flowrate
- GCU BOG consumption
- Auxiliary boiler diesel consumption

### 2.4 Emissions Modeling

Once the voyage was completed, the emissions measurement and ancillary data were synthesized and used to produce a multiparametric emissions model of methane and CO_2_ emissions from the roundtrip voyage. First, stack gas emission rates for methane and CO_2_ (in kg/h) were determined from the concentration measurements using the standard US EPA Method 19.^15^ Mass emissions of methane and CO_2_ were determined from the stack gas concentrations measured using FTIR spectroscopy (ppm or %v/v), stack gas moisture and O_2_ concentration (% v/v), fuel mass consumption rate, oxygen-based dry-basis fuel *F* factor (Sm^3^/J), and fuel heating value (mJ/kg).

Two of the six engine exhausts were continuously monitored at any one time, so an emission model was developed to estimate total emissions across the voyage. The correlation between ship operational/weather data and estimated engine exhaust emissions was investigated across all parameters detailed in the auxiliary data, mentioned in Section 2.3, and those that demonstrated a correlation were used to develop a parameterized model of emissions:

- Engine gas mode (gas mode and diesel mode)
- Load (% of maximum)
- Exhaust temperature (°C)
- BOG consumption (kg/h)
- Diesel consumption (kg/h)

Further information on the correlation factors and modeling to estimate total methane and CO_2_ emissions is given in the Supporting Information (Section 3 and Table S4). Normalized estimates of methane and CO_2_ emissions were estimated per tonne of LNG delivered: 67,500 tonnes of LNG was delivered at the unloading terminal in Zeebrugge.

## 3 Results

Results of the measurement study are first presented by describing total GHG emissions and then split by the voyage stage, by the GHG (methane or CO_2_), and by the emissions source to characterize the emissions profile. A technical summary of the voyage conditions, durations, fuel use, and emissions is given in the Supporting Information (Table S2), and the raw emissions data collected across the voyage can be found in the Supporting Information as a separate database.

### 3.1 Total Methane, CO_2_, and GHG Emissions

The total quantities of CO_2_ and methane emitted across the voyage were 4600 t CO_2_ and 68.1 t CH_4_, respectively. This equates to total GHG emissions of 7050 t CO_2equiv_ using a global warming potential (GWP) of 36 (100 year time horizon)^16^ or 10,500 t CO_2equiv_ using a GWP of 87 (20 year). These GWP values reflect the IPCC fifth assessment report including indirect warming effects.^1^ Expressed per unit of LNG delivered (67,500 t LNG delivered), GHG emissions are 104 g CO_2equiv_/kg LNG using GWP100 or 156 g CO_2equiv_/kg LNG using GWP20.

**Figure 1:**
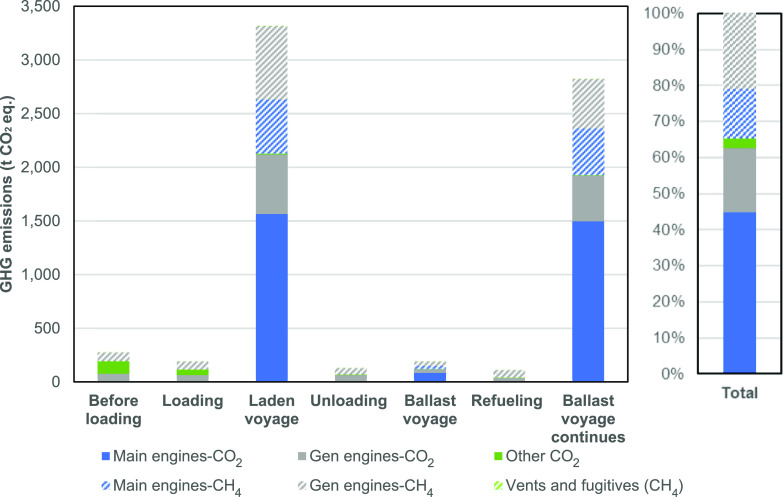
GHG emissions from different voyage segments, split by the emission source, using a GWP100 of 36. “Other CO_2_” includes CO_2_ emissions from the GCU and from the auxiliary boiler.

As shown in Figure 1, emissions are dominated by CO_2_ emissions, both from the main engines (45% of total GHGs) and the generator engines (18%). Methane emissions contribute 35% of the total GHG using GWP100. Using GWP20, this dominance is switched, and methane contributes 56% to the total GHG (as shown in Figure S1 in the Supporting Information).

Relating to methane emissions, the 68.1 tCH_4_ emissions equate to 0.1% of delivered LNG. The generator engines produce 60% of total methane emissions, compared with the main engines, which contribute 39%, as shown in the Supporting Information (Figure S2). Venting emissions (including maintenance activities) and fugitive emissions are non-zero but represent a minor proportion of the total for this ship voyage (160 kg CH_4_ or 0.23% of methane emissions).

### 3.2 Engine Methane Emissions

**Figure 2:**
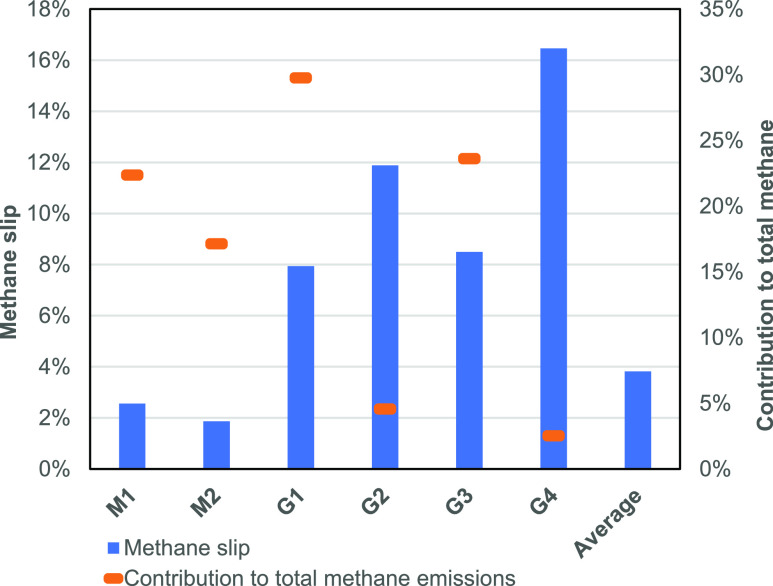
Methane slip rates (left axis), expressed as a percentage of LNG throughput, and their contribution to total methane emissions (right axis) across each engine.

The engines (both main and generator engines) contributed a total of 51.6 t methane emissions over the voyage, equating to 99.8% of total methane emissions. The generator engines have a substantially lower power output than the main engines (11,530 kW for each of the main engines, compared to 3840 and 2880 kW for the two generator engine types), but methane slip is substantially higher. Figure 2 shows the average methane slip rates for each engine (M_X_ = main engine and G_Y_ = generator engine) alongside the weighted average (in terms of the total voyage duration) across all engines.

The main engines exhibited average slip rates of 2.3 and 1.9% for M1 and M2, respectively. G1 and G3 were normally in operation (96 and 97% of the time, respectively, as per Table S1) and were preferentially used over G2 and G4 owing to operational issues with one of these engines. Average slip rates were 7.9 and 8.5% for G1 and G3, respectively. For emphasis, this means that ∼8% of all LNG that is sent into the generator engines slips out and is emitted directly to the atmosphere. G2 and G4 were only in operation for 14 and 5% of the voyage, respectively, but produced substantially higher slip rates. The variation in engine slip rates is explored further in the Discussion section.

### 3.3 Venting and Fugitives

Venting of natural gas was extremely low over the duration of the voyage. The operational philosophy includes zero routine venting for the storage system, and the only venting that occurred were due to the following reasons:

- fuel switching between gas and diesel modes for each of the engines, where the pipework was vented automatically as part of the changeover routing, and
- testing and maintenance activities where venting was required to isolate short sections of the pipework.

All engines were operated in the gas mode for >95% of the time, as detailed in Supporting Information Table S5. Engines were only in the diesel mode during engine start up, when operating at very low loads (typically less than 20%), or during the cleaning of the turbochargers, which occurs once every 200 h. Venting due to fuel switching occurs via an automated fuel switching process, where a section of the line (∼40 m length) is vented and purged with nitrogen to avoid creating an explosive mixture. Over the course of the voyage, the six engines were switched a total of 86 times, causing 47 kg CH_4_ emissions or 0.07% of total methane emissions measured as part of this study.

Maintenance practices minimized venting emissions by isolating short sections of the pipe, pressurizing with nitrogen and back-purging two–three times before venting low concentrations of methane. The measurement team monitored several operations and developed an inventory of testing and maintenance activities alongside the operational crew to determine what is maintained and how frequently. The estimate of total venting emissions for testing and maintenance over the duration of the voyage was negligible (<1 kg CH_4_).

During loading and unloading, a small amount of venting occurred to connecting/disconnecting the loading arms prior to LNG transfer. This again involved multiple nitrogen purges and back-flowing to the storage. The methane concentrations of gas within the short sections of the isolated pipe were tested via small vents prior and were less than 1 % vol/vol before disconnecting. Estimates of methane emissions were again less than 1 kg CH_4_, accounting for the volume, pressure, and temperature of LNG. One emission source during unloading that was detected but not possible to be quantified was a contaminated nitrogen vent on the loading arms. Nitrogen from onshore was used to blanket the flexible joints of the loading arms, and there was a slow vent discharging close to the loading arm connection. Investigators detected methane concentrations within this nitrogen purge in two out of the four loading arms, but there was insufficient time to quantify prior to disconnection. Again, this was perceived to be a negligible emission compared to engine slip.

For fugitive emissions, only two leaks were found over the duration of the trip. Both were continuous but negligible leaks. They were identified using the OGI camera, and then, an attempt to quantify was made using a high flow sampler. One leak was from a flanged pipe connection on the BOG delivery line to the generator engines and the other was from a pressure transmitter on the larger fuel delivery line from the compressor station to the engine room. However, the minimum detection limit for the high flow sampler was not reached for either leak, suggesting that each leak was lower than the stated minimum detection limit of 1.4 L/min. For the purposes of the calculation, 1.4 L/min flow was conservatively assumed for each of these leaks.

Both leaks sources were fixed onsite via the tightening of flanged connections. For emission quantification, it was assumed that these leaks occurred for the duration of the voyage. Though this was not the case (they were fixed in situ), these small emissions would have persisted if the investigators were not onboard, and so, this would have reflected the emissions of the voyage without manipulation from the investigators. Total fugitive emissions across the duration of the voyage were estimated to be 95 kg CH_4_, equating to 0.14% of total methane emissions computed as part of this study.

### 3.4 Other Sources of GHG Emissions

Other sources of GHG emissions that were studied represented a low contribution to total GHG emissions of 3%, as shown in Figure 1. These other sources were as follows:

- GCU (both methane and CO_2_)
- Auxiliary boiler (CO_2_)
- Reliquefaction plant (methane)

Besides the start of the voyage, where the GCU was operating briefly, BOG generation did not reach sufficient levels to require starting up of the reliquefaction plant or the GCU. The GCU is used when BOG generation is greater than the engine consumption for short periods of time, where the BOG is combusted and the exhaust gases are released to the atmosphere. The reliquefaction facility is operated when there is expected to be excess BOG generation for longer durations, for example, when the ship has stopped moving at the port and the main engines are switched off. It was noted by several engineers onboard that BOG generation is very low with current insulated LNG storage techniques and the ship can operate much more efficiently than with older systems. Earlier generations of LNG carriers, designed and constructed using older technologies for storage and BOG management, may exhibit varying emissions profiles compared to this ship.

## 4 Discussion

Further analysis of key emission sources is conducted in this section with a focus on the variation in methane slip, a comparison of GHG with other literature sources, identifying potential emission reductions going forward and improving methane measurements on ships.

### 4.1 Methane Slip versus Engine Load and Temperature

**Figure 3:**
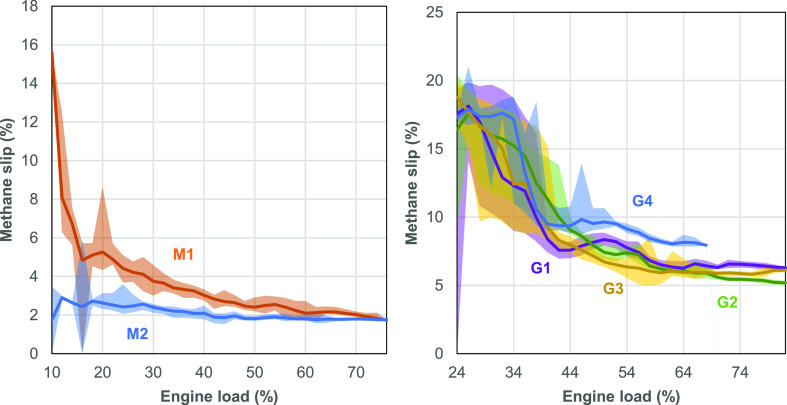
Methane slip by engine load across different main engines (left) and generator engines (right). Lines represent the mean, and shaded areas represent 5th–95th percentile values across measured data.

Methane slip rates are higher across the generator engines than anticipated in a previous research,^12^ and high variability was also demonstrated in the results: for example, generator engine G4 produced 16% slip (the uncombusted methane as a percentage of the methane throughput of the engine), double that of G1 (8%, which is the same model and power rating). Across all engines, methane slip changes with different engine loads, as shown in Figure 3.

The effect of engine load on slip is more pronounced for the LDPF four-stroke engines (generators) than for the LPDF two-stroke (main) engines, which exhibit a flatter load profile. All the generator engines exhibited a relatively similar slip across the load range, as shown in Figure 3, but the two main engines show significantly different slip rates across loads. M2 exhibited lower methane slip across all engine loads, with an average of 2% at higher loads (>50%) compared to 2.3% for M1.

In addition to engine load, methane slip also varied substantially across engine exhaust temperatures, and these correlations are shown in the Supporting Information (Figure S3). Exhaust temperature is governed by the air-to-fuel ratio, where lower methane slip rates were associated with higher exhaust temperatures or lower air-to-fuel ratios. This correlation was substantially stronger with generator engines, which exhibited a higher exhaust temperature than the main engines: main engine exhaust temperatures were typically 200–250 °C compared to those of the generators being 350–450 °C. This relationship between temperature, load, and methane slip is also reflected in the emission model parameters detailed in the Supporting Information (Table S4).

While methane slip across the engines was highly variable, the engines performed similarly to the manufacturer’s specifications or pre-engine tests. For the main engines, methane slip rates were broadly in line with the manufacturer’s specification, but there appears to be a larger deviation from the specification, particularly for M1 at lower engine loads. For the generator engines, methane slip was very similar to the pre-testing performance. Please see Supporting Information Section 4 for further details.

### 4.2 Comparison with Other Studies

**Figure 4:**
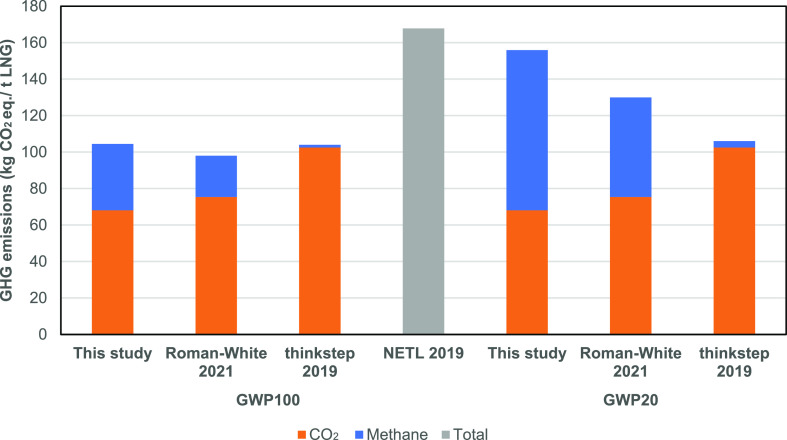
Comparison of GHG emissions from LNG shipping with other studies, expressed in kg CO_2equiv_/t LNG delivered, using both GWP100 and GWP20 climate metrics for methane. Source: refs (7)(17), and (18).

To understand the GHG results in context, this study is compared to three recent desk-based studies, Roman-White et al.,^7^ NETL,^17^ and thinkstep,^18^ as shown in Figure 4. All studies assess the life cycle GHG emissions associated with exporting LNG to different locations and included the LNG shipping transport as one part of the supply chain. Roman-White et al. modeled all cargoes delivered from Sabine Pass, USA, in 2018, the results of which this study compares with the average of all XDF ships from the USA to the UK as it closely matches the transport distance and propulsion technologies from this study. The NETL study comparison is for the USA—Rotterdam case study voyage using a DFDE vessel. The thinkstep study uses data from a previous NGVA study^19^ using fuel and emission factors for different LNG carrier types per megajoule kilometer (a megajoule of embodied energy in the LNG transported multiplied by the distance travelled): we took the emission factors for a 174,000 m^3^ DFDE vessel and applied our data to the distance and LNG delivered.

On a GWP100 basis, this study has similar GHG emissions to those of Roman-White et al. and thinkstep within a 7% range, but 38% lower than the NETL estimate. On a GWP20 basis, this study is 20–47% higher than the comparators.

The figure indicates the cause of some of this variation: the split between CO_2_ and methane emissions. Compared to the Roman-White 2021 and thinkstep studies, CO_2_ emissions from this study are 10–33% lower. For the NETL study, there was insufficient information on the split between CO_2_ and methane to compare. The lower CO_2_ emissions in this study arise from a lower fuel consumption than that assumed in all the studies, perhaps due to more efficient engine operation in modern gas engines and more efficient hull design. BOG consumption in this study was 88 kg/km (47.5 kg/Nm) and diesel consumption was 1.3 kg/km (0.7 kg/Nm), allowing for all uses of gas and diesel. The different engine technologies have varying efficiencies, and the LPDF two-stroke engine measured in this study is one of the most efficient marine gas engines.^12^

However, methane emissions over this voyage were 60% higher than those assumed by Roman-White et al.,^7^ which can be attributed to the difference in the assumed methane slip. The Roman-White study assumes a methane slip rate of 2.3%, which is comparable to that of our propulsion engines, whereas the average slip rate across all engines (including generators) was 3.8% in this campaign. The thinkstep study includes negligible methane slip rates with little justification and makes no mention of venting or fugitives but includes comparatively higher CO_2_ emissions, resulting in little variation across GWP time horizons.

The comparison with other studies has several limitations in terms of comparing “like-for-like”, primarily relating to transport distances and the propulsion technology. Similar distances (within 1000 km) were used, but only the Roman-White study^7^ used a comparable propulsion technology. Note that no other study explicitly modeled generator engine emissions, only the main propulsion or an aggregate (e.g., based on the total fuel consumption as per ref^7^). Studies also typically have not included emissions from venting or fugitives. This study demonstrates the importance of considering all emission sources to better understand the magnitude of emissions and to determine where the greatest reductions lie.

### 4.3 Emission Reductions Going Forward

Total emissions are similar for this ship compared with previous studies, but the contribution from methane is higher, and this represents a significant opportunity for further study and mitigation. Methane slip contributed 99% of methane emissions across the voyage and 35% of total GHG emissions (GWP100).

**Figure 5:**
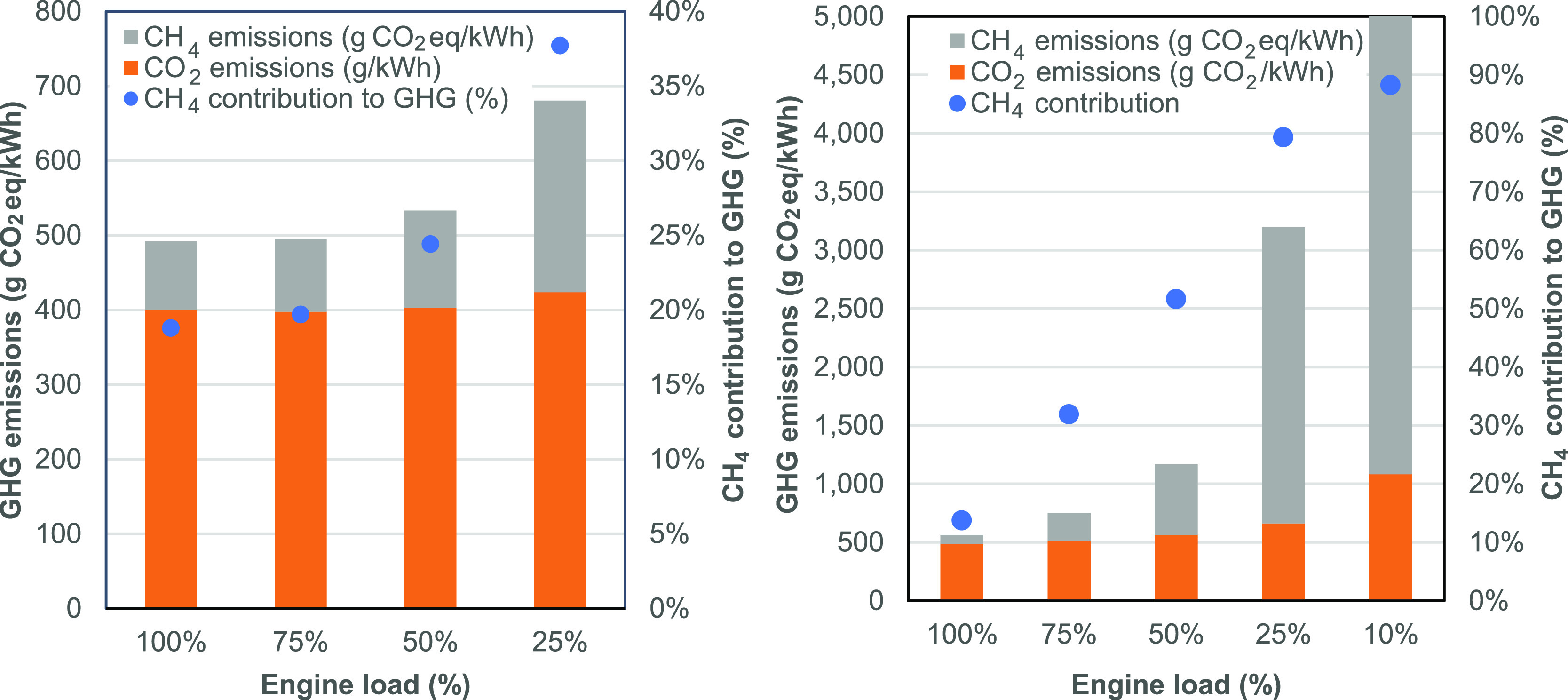
GHG emissions associated with different engine loads for main engine 1 (left) and generator engine (eight cylinder, right), split by the contribution from methane (GWP100) and CO_2_. The proportional contribution to total emissions from methane is indicated via the blue dots and the secondary axis.

During this campaign, the average engine load was approximately 40% across both the main and generator engines. This is lower than what may be considered optimal from an overall fuel use energy efficiency perspective but gives substantially higher methane slip as well. Figure 5 shows the total GHG emissions across different engine loads per unit of engine output for the generator (left) and main (right) engines. The graph demonstrates the exponential increase in methane emissions and its contribution toward GHG emissions with lower loads. Note that life cycle GHG emissions associated with using marine diesel oil are approximately 700 g CO_2equiv_/kW h engine output,^12^ which would require engine loads of above 75% to match for the generator engines but is improved upon at all engine loads for the XDF main engine.

It is not known whether operating at 40% load for generator engines is a typical mode of operation across the LNG carrier fleet, and discussions with LNG carrier operators suggest that they can, and do, operate at higher loads, which would reduce slip. For context, engine performance testing is conducted over a typical range of engine loads, which is on average 70% for constant-speed propulsion engines (E2 test cycle) and 47% for constant-speed auxiliary engines (D2 test cycle).^20^

The ship in this study was new, and this was only the ship’s second voyage, and so, operators preferred to have two engines in operation at any one time to avoid any shutdowns in case one engine stopped suddenly. However, if engines were operated closer to ∼80% load, methane emissions would have been reduced by over half (see Figure 5). Engine operational practices account for a broad range of safety, reliability, economic, and other environmental constraints, and further work in understanding how methane emission reduction opportunities could fit within these constraints is needed.

There are several additional opportunities to reduce methane slip, from the operational, exhaust treatment, and engine design perspectives. It should be noted that for the main propulsion engines, a new version of this XDF engine is now being offered, and it is suggested to reduce methane slip by approximately half, primarily owing to exhaust gas recirculation. There is an urgent need to identify and implement further feasible methods of methane emission reduction if natural gas is to contribute to meeting maritime GHG reduction targets.^21^

### 4.4 Improving Methane Measurement on Ships

A better understanding of methane emissions and their sources/causes on different ships is required to identify the best methods to reduce emissions. Several questions arise from this study, including the following: What are the real-world methane emissions across different LNG ships; how do emissions vary by ship age, cargo tank and insulation technology, size, operator, and engine type; and are venting and fugitive emissions similarly small for other ships? There are two important next steps to better understand and reduce methane emissions: a broader independent measurement study to provide a robust understanding of fleet-wide methane emissions; and increased industrial self-monitoring of methane emissions.

More independent, published measurements of methane emissions from LNG carriers and shipping more broadly are needed to obtain a representative sample and to gain an understanding of how to mitigate emissions. It is important to emphasize that this single measurement campaign does not constitute a representative sample of the LNG carrier fleet, which constituted 572 active vessels in 2021.^13^ Similar to other segments of the natural gas supply chain, methane and CO_2_ emissions vary by region, operator, technology, and age of facility among other factors. To understand the climate impact of importing LNG from different regions, we need to understand how methane emissions vary, and a representative sample of measurements is required across the different types of engines, storage technologies, ships, and operators. This will enable a greater understanding of not only emissions but also how they can be reduced most cost effectively and by how much.

This study employed a bottom-up engineering approach to measurement, where we first assessed all potential emitting sources and then attempted to measure/screen every potential source: FTIR continuous emissions monitoring for the stack emissions, OGI camera leak detection for fugitives, and a combination of ship ancillary data, operator expertise, and OGI camera spot checks for venting emissions. This campaign was particularly comprehensive in its scope for fugitive emissions detection, where each unit was reviewed around five times over the course of the voyage: OGI surveys for many other oil and gas facilities may occur only once every year. The advantage of this measurement methodology is that by studying emissions from all known individual sources, we were able to collect sufficient data to understand where the emissions arise from and what their causes were. This is vital in efforts to reduce emissions going forward.

The experimental design aimed to ensure that there were no unaccounted emission sources by identifying all potential leaks and with regular surveying, but a general disadvantage of bottom-up measurements is that we are unable to confirm the absence of “unknown unknowns”, that is, potential emission sources that were not identified during planning or during the measurement campaign. Top-down studies using aircrafts or drones could be useful as a cross-sectional snapshot of emissions across several ships and could help ensure that all emission sources are accounted for via a reconciliation study between top-down and bottom-up methods.^22^

Moving beyond these independent measurement studies that provide a robust emissions baseline, it is important for the industry to self-monitor methane emissions. In the absence of regulation on methane emissions from shipping, increased in-house monitoring of methane emissions would provide assurance, help further the understanding of emissions, and drive down emissions. There are several CEMSs that are commercially available for methane in engine exhaust. These have been installed on some ships with gas engines (the authors are aware of several ships with installed monitoring), but the extent to which these are used is unknown. Additionally, to assess and reduce fugitive emissions, it is recommended that ship operators are trained in using an OGI camera to monitor emissions periodically during voyage. A best practice guide on effective techniques and methods for monitoring methane emissions in shipping would be a valuable contribution toward greater industry participation, for example, via Methane Guiding Principles.^23^ In addition to techniques for measurement, it is important to maintain an inventory of methane emissions that can help us understand how they may be prevented in the future and to feed into continual improvement.

## 5 Implications

This study has demonstrated a successful bottom-up measurement campaign of an LNG carrier using industry-standard measurement systems that help determine the causes of methane emissions and shed light on how they may be mitigated in future. Variation in methane emissions with different operational profiles are highlighted here. During this voyage, all engines were operated at relatively low loads, averaging 40–45% across all engines. If the engines were kept at 80%, this would approximately halve methane emissions and result in much more efficient engine operation.

Already much variation in methane emissions has been highlighted, and it is likely that the highly heterogeneous LNG shipping fleet also exhibits variability in methane emissions across the range of ships with different ages, engine technologies, storage technologies, boil-off management, and operators. More studies like this must be conducted to develop a representative sample, to understand current emissions, and more importantly to develop cost-effective mitigation strategies. A further recommendation is for ship operators to install methane emissions monitoring on engine exhausts and to conduct periodic leak detection surveys to feed into methane-minimizing operational practices.

## Supporting Information Available

Additional details on the measurement and modeling methodology; results of total methane and CO_2_ emissions; methane slip correlation with temperature; and comparison of emissions with the manufacturer’s data (PDF)
Raw exhaust concentration data from FTIR CEMS A (XLSX)
Raw exhaust concentration data from FTIR CEMS B (XLSX)
