# Supplementary material for: Total Methane and CO2 Emissions from Liquefied Natural Gas Carrier Ships: The First Primary Measurements
Source: Environ Sci Technol. 2022 Jun 14;56(13):9632–40. doi: 10.1021/acs.est.2c01383 (PMC9261184; doi:10.1021/acs.est.2c01383)
Supplement: Supplementary file 1 — es2c01383_si_001.pdf [file es2c01383_si_001.pdf]

# Supplementary Information

Paper: Total methane and CO2 emissions from LNG carriers: the first primary measurements

Paul Balcombe[i*], Dalia A. Heggo[i], Matthew Harrison[ii]

[i]School of Engineering and Material Sciences, Queen Mary University of London, E1 4NS, UK
[ii]SLR Corporation, 22118 20th Ave SE, Bothell, WA 98021 , Bothell, Washington 98021 United States

* Corresponding author: p.balcombe@qmul.ac.uk

# pages: 11

# figures: 6

# tables: 5

# 1. Additional methodological detail

*Table SI-1. Voyage summary.*

| Voyage stage | Date | Location/ Distance |
|---|---|---|
| Before loading | 25th – 27th March | Corpus Christi, USA |
| Loading | 27th March | Corpus Christi, USA |
| laden voyage | 28th March – 13th April | 10,284 km |
| Unloading | 13th April | Zeebrugge, Belgium |
| Ballast voyage | 14th – 15th April | 609 km |
| Refuelling | 15th April | Portland, UK |
| Ballast voyage continued | 15th – 28th April | 9,318 km, to Corpus Christi, USA |

*Table SI-2. Descriptive statistics of the measurement data across different stages of the voyage.*

| | Before loading | Loading | Cargo Voyage | Unloading | Ballast voyage | Refuelling | Ballast voyage continues | Total |
|---|---|---|---|---|---|---|---|---|
| Duration (days) | 1.7 | 1.5 | 15.8 | 1.0 | 1.0 | 0.8 | 12.7 | 34.5 |
| Speed (knots) | 1.3 | 0 | 14.6 | 0 | 13.5 | 0 | 16.5 | |
| Speed (km/hr) | 2.5 | 0 | 27.1 | 0 | 24.9 | 0 | 30.6 | |
| Distance (km) | 99.4 | 0 | 10,283.8 | 0 | 608.8 | 0 | 9,318.1 | 20,310.1 |
| Distance (Nm) | 53.7 | 0 | 5,552.8 | 0 | 328.7 | 0 | 5,031.4 | 10,966.6 |
| Wind speed (m/s) | 7 | 8 | 10 | 3 | 5 | 6 | 9 | |
| Wind direction (deg) | 196 | 93 | 208 | 177 | 108 | 271 | 230 | |
| Ambient temperature (C) | 21 | 23 | 19 | 7 | 9 | 8 | 22 | |
| Average BOG consumption (kg/h) | 1,815 | 1,224 | 2,165 | 1,034 | 1,833 | 726 | 2,484 | 2,148 |
| Average oil consumption (kg/h) | 91 | 66 | 31 | 90 | 95 | 77 | 7 | 31 |
| M1 active (%) | 10% | 1% | 97% | 2% | 93% | 1% | 100% | 85% |
| M2 active (%) | 11% | 1% | 98% | 1% | 93% | 0% | 100% | 85% |
| G1 active (%) | 86% | 100% | 96% | 100% | 100% | 100% | 95% | 96% |
| G2 active (%) | 15% | 56% | 7% | 98% | 13% | 58% | 9% | 14% |
| G3 active (%) | 47% | 100% | 100% | 100% | 100% | 100% | 98% | 97% |
| G4 active (%) | 63% | 0% | 5% | 0% | 0% | 0% | 0% | 5% |
| M1 load (when on) % | 11% | 6% | 44% | 3% | 38% | 6% | 54% | 48% |
| M2 load (when on) % | 11% | 6% | 45% | 4% | 39% | 5% | 56% | 49% |
| G1 load (when on) % | 48% | 44% | 41% | 60% | 40% | 38% | 42% | 42% |
| G2 load (when on) % | 58% | 41% | 32% | 61% | 39% | 37% | 43% | 44% |
| G3 load (when on) % | 48% | 44% | 41% | 60% | 40% | 37% | 42% | 42% |
| G4 load (when on) % | 54% | - | 26% | - | - | - | - | 41% |
| M1 gas mode use (%) | 0% | 0% | 98% | 0% | 89% | 0% | 100% | 98% |
| M2 gas mode use (%) | 0% | 0% | 98% | 0% | 90% | 0% | 100% | 98% |
| G1 gas mode use (%) | 99% | 100% | 99% | 96% | 100% | 100% | 99% | 99% |
| G2 gas mode use (%) | 98% | 99% | 96% | 96% | 99% | 100% | 95% | 96% |
| G3 gas mode use (%) | 99% | 100% | 99% | 97% | 100% | 100% | 100% | 99% |
| G4 gas mode use (%) | 99% | - | 95% | - | - | - | 0% | 95% |

| | Before loading | Loading | Cargo Voyage | Unloading | Ballast voyage | Refuelling | Ballast voyage continues | Total |
|---|---|---|---|---|---|---|---|---|
| M1 total BOG (kg) | 0 | 0 | 295,678 | 0 | 15,097 | 0 | 288,776 | 599,551 |
| M2 total BOG (kg) | 0 | 0 | 310,203 | 0 | 15,825 | 0 | 300,541 | 626,569 |
| G1 total BOG (kg) | 12,098 | 11,800 | 116,000 | 10,028 | 7,550 | 6,639 | 90,433 | 254,548 |
| G2 total BOG (kg) | 1,830 | 4,939 | 5,819 | 7,595 | 721 | 3,084 | 6,604 | 30,593 |
| G3 total BOG (kg) | 4,668 | 8,597 | 89,742 | 7,359 | 5,571 | 4,833 | 69,156 | 189,925 |
| G4 total BOG (kg) | 9,433 | 0 | 5,263 | 0 | 0 | 0 | 0 | 14,696 |
| GCU total BOG (kg) | 44,530 | 17,510 | 60 | 0 | 0 | 0 | 85 | 62,185 |
| M1 methane slip (%) | - | - | 2.7% | - | 3.1% | - | 2.3% | 2.6% |
| M2 methane slip (%) | - | - | 1.9% | - | 2.1% | - | 1.8% | 1.9% |
| G1 methane slip (%) | 8.2% | 8.1% | 8.0% | 7.0% | 8.2% | 12.8% | 7.5% | 7.9% |
| G2 methane slip (%) | 6.4% | 9.9% | 15.1% | 6.5% | 10.6% | 14.3% | 9.2% | 11.9% |
| G3 methane slip (%) | 7.3% | 8.2% | 8.8% | 6.7% | 9.0% | 13.4% | 7.9% | 8.5% |
| G4 methane slip (%) | 8.6% | - | 17.3% | - | - | - | - | 16.5% |
| Total main engine slip (%) | - | - | 2.3% | - | 2.6% | - | 2.0% | 2.2% |
| Total generator engine slip (%) | 8.1% | 8.5% | 8.8% | 6.8% | 8.6% | 13.4% | 7.7% | 8.7% |
| Total methane emissions (t) | 2.26 | 2.15 | 33.00 | 1.69 | 2.00 | 1.94 | 24.90 | 67.9 |
| Methane emissions/ LNG delivered (%) | 0.003% | 0.003% | 0.049% | 0.003% | 0.003% | 0.003% | 0.037% | 0.10% |
| Methane emissions- main engines (t) | 0.0 | 0.0 | 14.0 | 0.0 | 0.8 | 0.0 | 12.0 | 26.8 |
| Methane emissions- generator engines (t) | 2.3 | 2.1 | 19.0 | 1.7 | 1.2 | 1.9 | 12.9 | 41.1 |
| Total engine GHG (t CO2eq) | 272.9 | 186.9 | 3,306.6 | 126.9 | 192.0 | 107.1 | 2,818.3 | 7,010.6 |
| CO2 emissions- main engines (t CO2) | 6.8 | 0.7 | 1,565.7 | 0.4 | 84.9 | 0.2 | 1,498.9 | 3,157.6 |
| CO2 emissions- generator engines (t CO2) | 71.7 | 64.4 | 552.8 | 65.6 | 35.2 | 37.0 | 422.8 | 1,249.4 |
| GCU CO2 emissions (t CO2) | 113.1 | 44.5 | 0.2 | 0.0 | 0.0 | 0.0 | 0.2 | 157.9 |
| Total vent emissions (kg CH4) | 6.6 | 1.1 | 24.1 | 8.2 | 4.9 | 0.5 | 19.2 | 65.1 |
| Fugitive emissions (kg CH4) | 4.6 | 4.0 | 43.4 | 2.8 | 2.8 | 2.3 | 34.7 | 94.5 |
| Aux boiler emissions (t CO2) | 4.1 | 6.7 | 8.1 | 4.3 | 0.9 | 4.7 | 4.0 | 32.9 |
| Total CO2 (t CO2) | 195.8 | 116.2 | 2,126.8 | 70.3 | 121.0 | 41.9 | 1,926.0 | 4,597.9 |
| Total methane (t CH4) | 2.3 | 2.2 | 33.1 | 1.7 | 2.0 | 1.9 | 25.0 | 68.1 |
| Total GHG (t CO2eq) | 277.4 | 193.7 | 3,317.1 | 131.6 | 193.2 | 111.9 | 2,824.2 | 7,049.3 |
| Total GHG (g CO2eq./ kg LNG) | 4.1 | 2.9 | 49.2 | 2.0 | 2.9 | 1.7 | 41.8 | 104.5 |
| Methane contribution (GWP100) | 29% | 40% | 36% | 47% | 37% | 63% | 32% | 35% |
| GWP20 total methane (t CO2eq) | 197.3 | 187.4 | 2,876.7 | 148.1 | 174.5 | 169.4 | 2,170.8 | 5,924.1 |
| Total GHG (GWP20 t CO2eq) | 393.1 | 303.6 | 5,003.4 | 218.5 | 295.5 | 211.2 | 4,096.8 | 10,522.0 |
| Total GHG (gCO2eq./ kg LNG) GWP20 | 5.8 | 4.5 | 74.1 | 3.2 | 4.4 | 3.1 | 60.7 | 155.9 |
| Methane contribution | 50% | 62% | 57% | 68% | 59% | 80% | 53% | 56% |

*Table SI-3. Sources of methane and $CO_2$ emissions from the roundtrip voyage.*

| Source | Methane | $CO_2$ |
|---|---|---|
| Main engine 1 (M1) | x | x |
| Main engine 2 (M2) | x | x |
| Generator engine 1 (G1) | x | x |
| Generator engine 2 (G2) | x | x |
| Generator engine 3 (G3) | x | x |
| Generator engine 4 (G4) | x | x |
| Gas Combustion Unit (GCU) | x | x |
| Auxiliary boiler | | x |
| Vent mast 1 (forward) | x | |
| Vent mast 2 | x | |
| Vent mast 3 | x | |
| Vent mast 4 | x | |
| Engine room vent | x | |
| Vents from loading/unloading | x | |
| Vents from maintenance activities | x | |
| Fugitives (all gas-handling equipment) | x | |

# 2. Measurement technologies and processes

## Additional information on the FTIR setup

Fourier Transfer Infrared Spectroscopy (FTIR) is an optical absorption technology. The extractive FTIR method was selected because it has been long and widely used for many years, including for reciprocating internal combustion engines, and the method (Method 320) has been promulgated by the U.S. Environmental Protection Agency (EPA) and codified into the U.S. Code of Federal Regulations. Furthermore, FTIR is well suited to measure $CH_4$ and $CO_2$.

Samples were extracted from each engine exhaust using a 19 mm diameter 316 stainless steel multi-hole probe (MHP) configured with sample holes located in accordance with US EPA Method 1 [1]. MHPs were installed at each of the six engine sample points and the GCU sample point. Heated sample lines were connected to the MHPs and directed to the FTIRs. A continuous sample was extracted from two exhaust stacks at 1-2 litres/min and routed to the MKS Model 2030 MultiGas™ FTIR Analyzer via the MHP, heated sample line and heated sample pump. Each sample line and sample pump was maintained at 121 °C to prevent condensation of the gas sample. The FTIR gas cell was maintained at 191 °C. A slipstream of the sample was routed to an $O_2$ analyser to quantify stack gas $O_2$ concentration (%v/v).

## Venting emissions

In addition to conducting spot checks of vent masts throughout the duration of the journey, an inventory of venting operations was developed to estimate total venting emissions using standard engineering calculations.

Firstly, interviews with the chief engineer and cargo engineer were conducted to identify all of the venting operations that occur as per their standard operating procedures. 8 different venting

operations were identified, relating to fuel switching of the main and generator engines, as well as several maintenance activities where isolated sections of pipework were vented.

1. Depressurisation of pipe prior to loading arm connection
2. Depressurisation of pipe prior to loading arm disconnection
3. Loading manifold filter check
4. Main and generator engine fuel switch from gas to diesel
5. Compressor room vapouriser filter check
6. Reliquefaction filter check
7. Spray line filter check
8. Vent mast control valve check

For every venting instance, information was recorded relating to: frequency of operation (# per voyage); volume of vented equipment; operating pressure and temperature; and gas composition. This data was used to estimate the total volume of methane emissions for each event. Standard operating procedures were to pressurise the isolation section with nitrogen to 5 bar, then typically back purge to LNG storage several times prior to checking the composition of the volume was lower than 1% methane (~20% of the lower explosive limit). The ideal gas law was used to estimate the venting volume at Normal conditions, which was then multiplied by the number of operations per roundtrip voyage. The researchers witnessed each maintenance operation to confirm the calculation was accurate.

### Fugitive emissions

For the cargo containment system, the OGI detection is limited by wind speeds which were variable. However, all equipment was accessible to within ~2m and consequently it was deemed to be an appropriate leak detection method.

### Ancillary data

During the voyage the investigators held daily meetings with the captain and lead engineers to discuss measurement strategies, understand what activities were being conducted on that day, to identify safety requirements for that day and to collect ancillary data from the ship.

## 3. Further information on engine modelling

When the engines were operating on diesel mode, methane emissions are assumed to be zero. In reality there are likely to be low levels of methane emissions but these are orders of magnitude lower than whilst operating on gas mode [2, 3].

Several regressions with different parameters were conducted to understand the relationship between methane emissions and operational parameters. Methane emissions were converted into methane slip, expressed as a percentage of BOG flow to the engine that slips out uncombusted, as it has been previously asserted that methane slip is heavily affected by engine load [4]. Various linear, log-linear and log-log relationships were assessed across different combinations of ancillary parameters, as well as allowing for potential correlation between the independent variables. Each engine was regressed separately, and all engines found the best fit regressions to be based on:

$$M(\%) = \alpha + \beta_1 \log_{10}(L(\%)) + \beta_2 T(°C)$$

where $M$ is the methane slip as a % of BOG consumption, $L$ is the engine load as a % of maximum capacity, $T$ is the exhaust temperature in °C, $\alpha$ is the coefficient constant, $\beta_1$ and $\beta_2$ are the

coefficients associated with load and exhaust temperature, respectively. A table of coefficient values are included in the SI Table SI-2.

$CO_2$ emissions are directly correlated with fuel consumption and are governed by combustion efficiencies [5]. Measured $CO_2$ emissions were regressed against fuel consumption in aggregate and the following relationship was used to estimate $CO_2$ emissions:

$$C\left(\frac{kg}{hr}\right) = 2.54\left(\frac{kgCO_2}{kg}\right).BOG\left(\frac{kg}{hr}\right) + 3.16\left(\frac{kgCO_2}{kg}\right).Diesel\left(\frac{kg}{hr}\right)$$

where *C* is the $CO_2$ emission rate in kg/hr, *BOG* is the BOG consumption in kg/hr, and *Diesel* is the diesel consumption in kg/hr. This closely aligns with standard models of $CO_2$ emissions from fuel combustion [5, 6].

The estimation of total methane and $CO_2$ emissions is the sum of all sources across the voyage.

## LNG composition

The composition of the LNG cargo was: 96% methane, 3.8% ethane, plus other trace components. In estimating the methane slip rate (as a percentage of gas used as fuel) it was assumed that the fuel used was pure methane. The boil-off used for the engines is likely to have a slightly higher concentration of methane but the composition was not directly monitored. The impact of this composition variation on the calculations were deemed to be minimal.

# 4. Additional results

*Table SI-4. Engine emissions model coefficients, relating to equation X. VIF = variance inflation factor which is a measure of the collinearity between parameters, in this case correlation between log(load) and temperature. Values of 1 indicate no correlation and above 5 indicate strong correlation.*

| Engine | Intercept | log(load) | temp | $R^2$ | VIF |
|---|---|---|---|---|---|
| M1 | 17.6 | -2.6 | -0.022 | 0.73 | 1.22 |
| M2 | 8.2 | -0.82 | -0.014 | 0.47 | 1.32 |
| G1 | 71.6 | -6.8 | -0.086 | 0.84 | 1.25 |
| G2 | 62.7 | -8.4 | -0.051 | 0.88 | 1 |
| G3 | 73.3 | -7.8 | -0.083 | 0.93 | 1.21 |
| G4 | 57.3 | -4.7 | -0.069 | 0.55 | 1 |

*Table SI-5. Summary statistics of engine performance for each engine.*

| | M1 | M2 | G1 | G2 | G3 | G4 |
|---|---|---|---|---|---|---|
| Engine load (%) when active | 42% | 44% | 42% | 39% | 42% | 29% |
| Time active (%) | 85% | 85% | 96% | 14% | 97% | 5% |
| Gas mode (%) | 98% | 98% | 99% | 96% | 99% | 95% |
| BOG consumption (kg/hr) | 870.6 | 909.3 | 325.0 | 245.0 | 238.3 | 304.6 |
| Methane slip (%) | 3% | 2% | 8% | 12% | 8% | 16% |

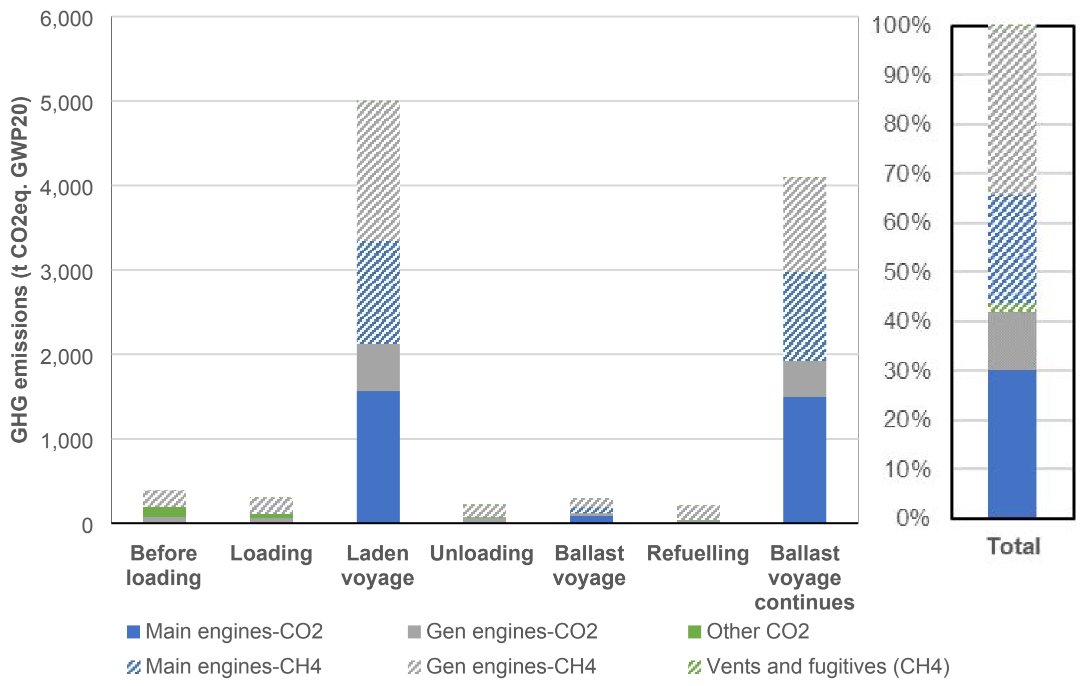


*Figure SI-1. Total greenhouse gas emissions from the roundtrip voyage using a GWP20 of 87.*

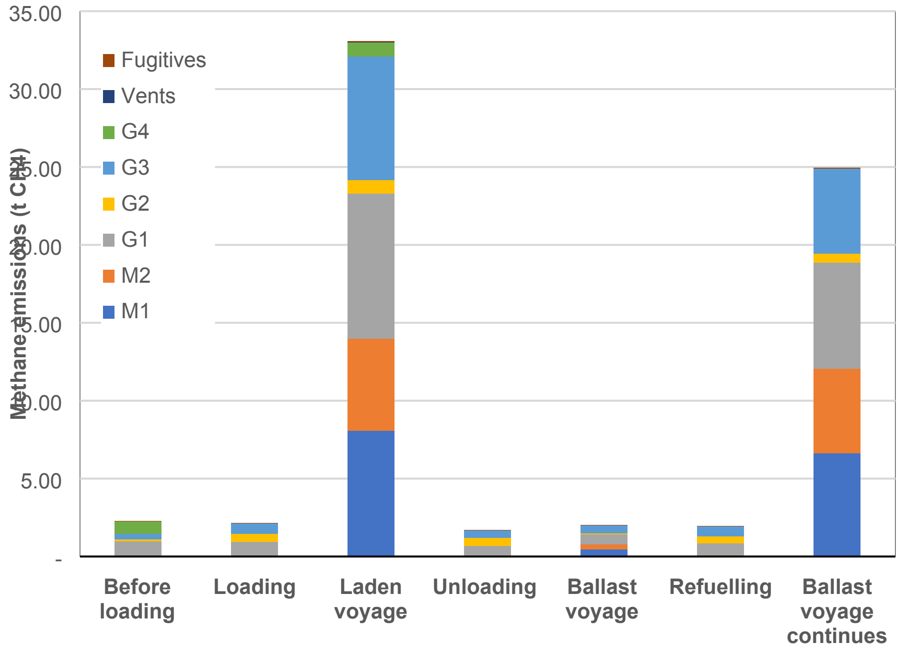


*Figure SI-2. Methane emissions from different voyage segments, split by methane emission source.*

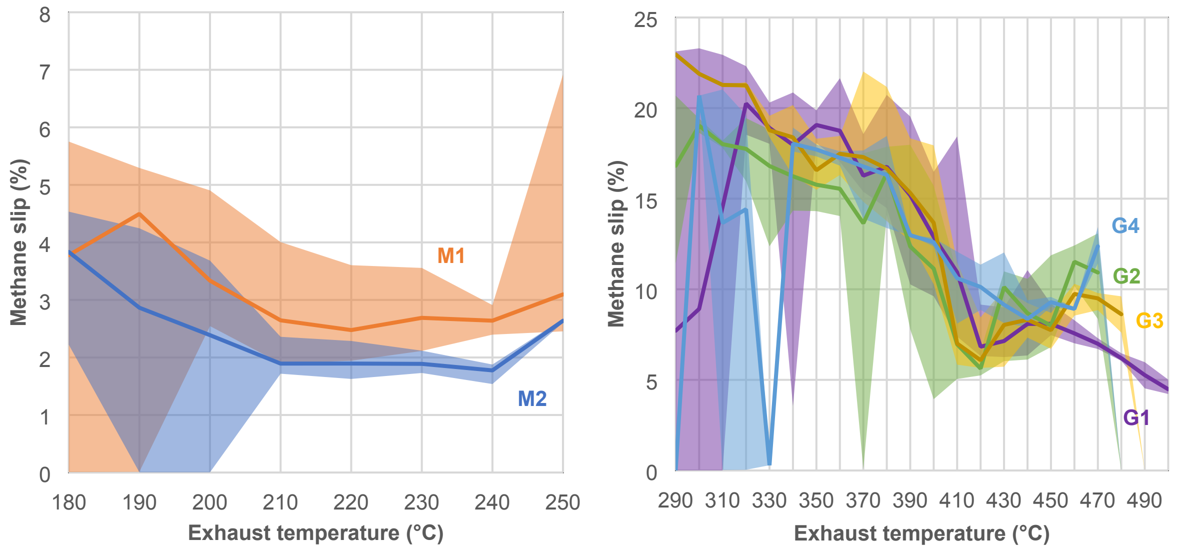


*Figure SI-3 Methane slip rates over varying exhaust temperatures for main engines (left) and generator engines (right). Solid lines indicate the mean average values and the shaded areas represent the 5th - 95th percentile range for each engine*

## Methane slip vs manufacturers' specification

Whilst methane slip across the engines was highly variable, the engines performed similarly to the manufacturers specifications or pre engine tests. This can be seen in Figure  which compares average slip rates across engine loads for the main engines (left) and the generators (right), with manufacturers data (depicted as dotted lines). For the generator engines, the factory test results ('NOX technical file') were used. For the main engines, data was supplied by the manufacturer WinGD as there was insufficient information within the 'NOX technical file' to conduct this calculation.

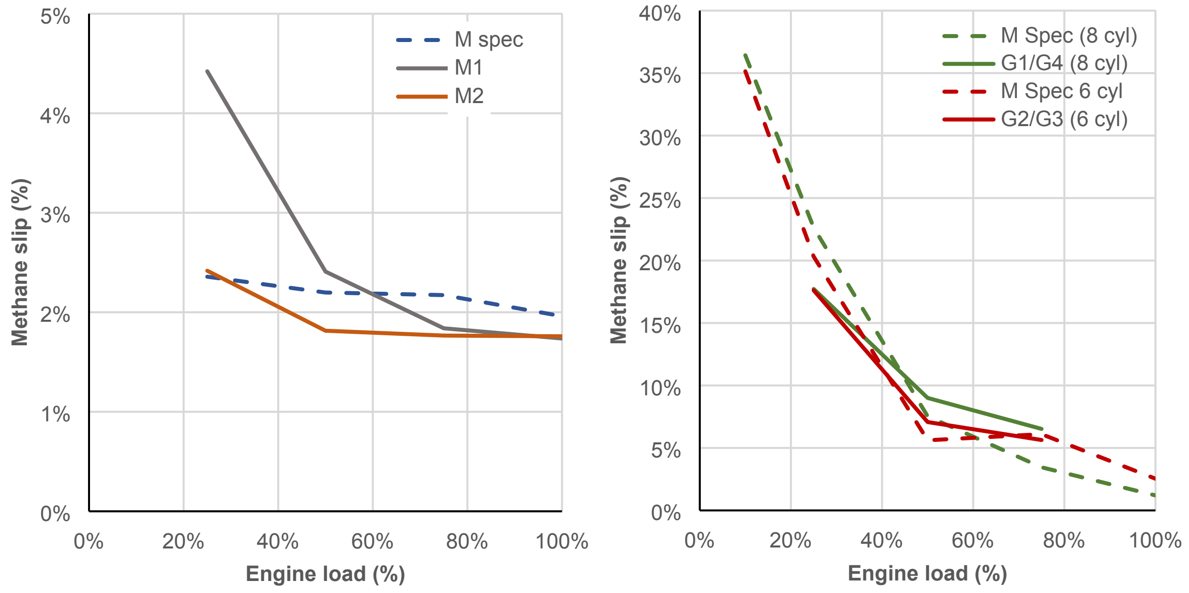


*Figure SI-4. Methane slip across engine load compared with manufacturer specifications ('M Spec', dotted lines). Main engines (left) and generator engines (right, split by size)*

For the main engines, methane slip rates were broadly in line with the manufacturer's specification, but there appears to be a larger deviation from the specification, particularly for M1 at lower engine loads. For the generator engines, methane slip was very similar to the pre-testing performance. Note that the scale of methane slip on the y-axis from the main engines are an order of magnitude lower in the graphs than for the generator engines, so whilst the main engines may appear more variable the differences in main engine performance do not result in significant differences in methane emissions.

## Comparison with other engine slip rates

Out of the two engine types monitored in this study (LPDF 2-stroke for propulsion and LPDF 4-stroke for the generators) there is substantially more primary measurement for the LPDF 4-strokes and a comparison is made with other literature estimates in the graphs below. For the LPDF 2-strokes, only the manufacturers information is currently available but this suggests that the engines were operating within the expected performance (average of ~2% slip). For the LPDF 4-strokes, there are several estimates that were collated in a previous study[2] and supplemented with additional data points[7, 8]

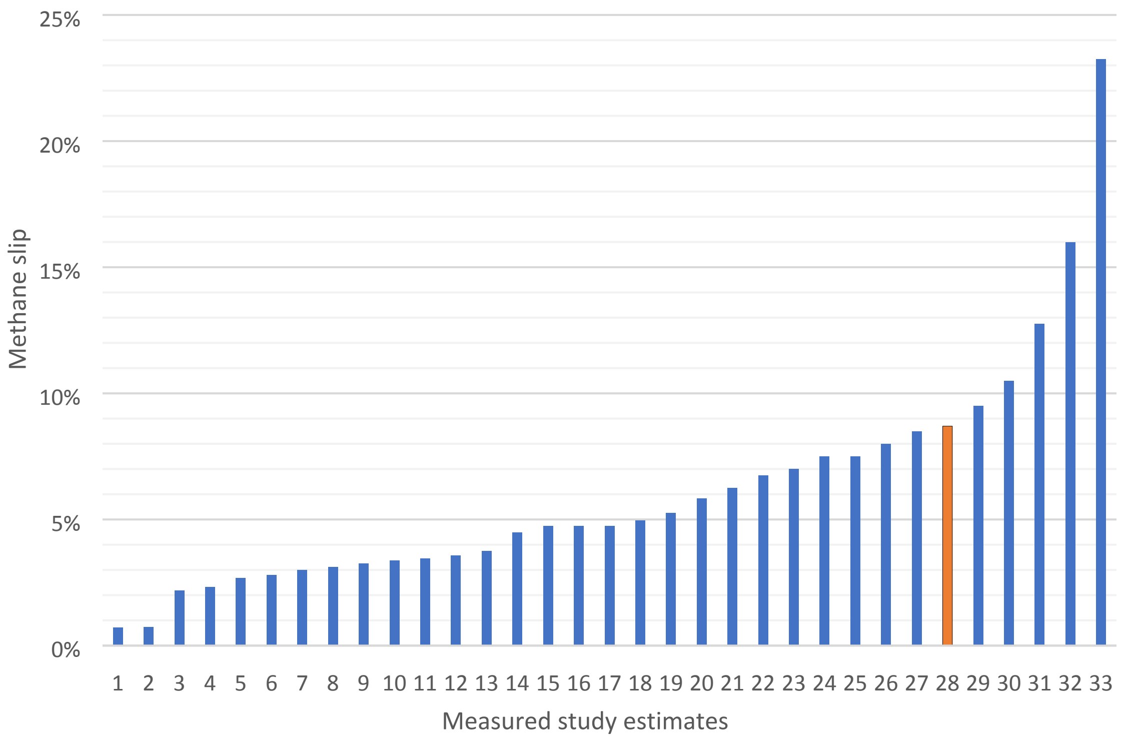


*Figure SI-5. Comparison of methane slip measurements for LPDF 4-stroke marine engines. The orange (with black border) bar represents the average LPDF 4-stroke slip rate in this study. Source:* [2, 7, 8]

A comparison of the change in methane slip with engine load is made in the following figure, comparing measurements of an LPDF 4-stroke marine engine from Sommer et al. [4] with the G1 and G3 generator engines in this study. Slip rates are relatively similar, particularly across lower loads, although it appears that higher loads exhibit higher emissions in our study compared to the Sommer et al. study.

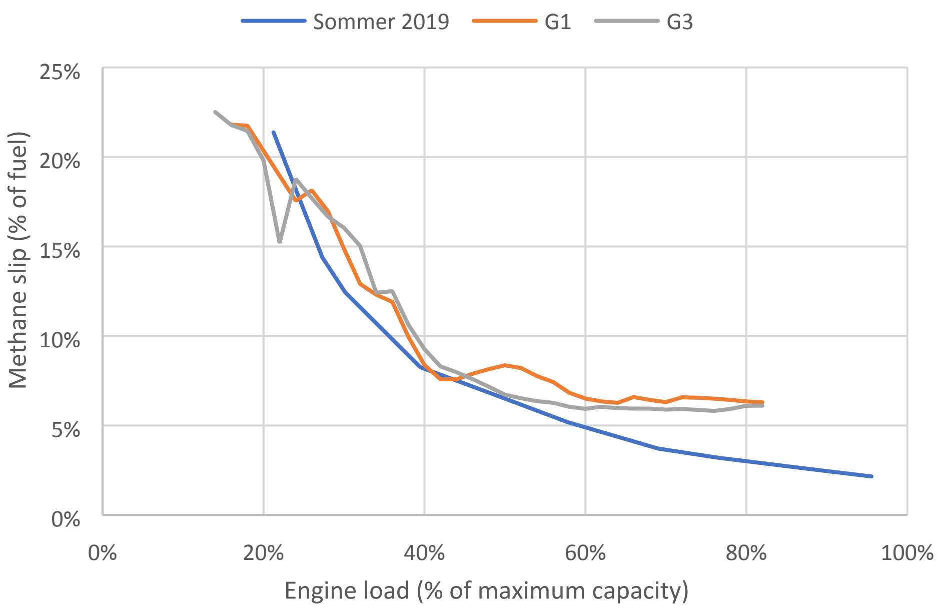


*Figure SI-6. Comparison of methane slip by engine load with the Sommer et al. 2019 study of an LPDF 4-stroke marine engine, compared with engines G1 and G3 from this study.*

# References

1. US EPA *Method 1 - Sample/ Velocity Traverses*; US Environmental Protection Agency: Accessed from: www.epa.gov/sites/default/files/2020-08/documents/qa_vol_iii_-_sept_1994_m1.pdf, 1994; pp 1-8.
2. Balcombe, P.; Staffell, I.; Kerdan, I. G.; Speirs, J. F.; Brandon, N. P.; Hawkes, A. D., How can LNG-fuelled ships meet decarbonisation targets? An environmental and economic analysis. *Energy* **2021,** *227*, 120462.
3. Stenersen, D.; Thonstad, O. *GHG and NOx emissions from gas fuelled engines. Mapping, verification, reduction technologies*; SINTEF Ocean AS: Trondheim, Norway, 2017; pp 1-52.
4. Sommer, D. E.; Yeremi, M.; Son, J.; Corbin, J. C.; Gagné, S.; Lobo, P.; Miller, J. W.; Kirchen, P., Characterization and Reduction of In-Use CH4 Emissions from a Dual Fuel Marine Engine Using Wavelength Modulation Spectroscopy. *Environmental Science & Technology* **2019,** *53*, (5), 2892-2899.
5. UK BEIS *2021 Government Greenhouse Gas Conversion Factors for Company Reporting. Methodology Paper for Conversion factors*; UK Government Department for Business, Energy and Industrial Strategy: Accessed from: https://assets.publishing.service.gov.uk/government/uploads/system/uploads/attachment_data/file/990675/2021-ghg-conversion-factors-methodology.pdf, 2021; pp 1-133.
6. IMO *Annex 5, resolution MEPC.245(66): Guidelines on the method of calculation of the attained Energy Efficiency Design Index (EEDI) for new ships*; International Maritime Organization: Accessed from:

https://wwwcdn.imo.org/localresources/en/KnowledgeCentre/IndexofIMOResolutions/MEPCDocuments/MEPC.245(66).pdf, 2014; pp 1-32.
7. Anderson, M.; Salo, K.; Fridell, E., Particle- and Gaseous Emissions from an LNG Powered Ship. *Environmental Science & Technology* **2015,** *49*, (20), 12568-12575.
8. Grönholm, T.; Mäkelä, T.; Hatakka, J.; Jalkanen, J.-P.; Kuula, J.; Laurila, T.; Laakso, L.; Kukkonen, J., Evaluation of Methane Emissions Originating from LNG Ships Based on the Measurements at a Remote Marine Station. *Environmental Science & Technology* **2021,** *55*, (20), 13677-13686.
